# An innovative antenna array with high inter element isolation for sub-6 GHz 5G MIMO communication systems

**DOI:** 10.1038/s41598-022-12119-2

**Published:** 2022-05-12

**Authors:** Mohammad Alibakhshikenari, Bal S. Virdee, Harry Benetatos, Esraa Mousa Ali, Mohammad Soruri, Mariana Dalarsson, Mohammad Naser-Moghadasi, Chan Hwang See, Anna Pietrenko-Dabrowska, Slawomir Koziel, Stanislaw Szczepanski, Ernesto Limiti

**Affiliations:** 1 Department of Signal Theory and Communications, Universidad Carlos III de Madrid, 28911 Leganés, Madrid, Spain; 2 School of Computing and Digital Media, Center for Communications Technology, London Metropolitan University, London, N7 8DB UK; 3 Faculty of Aviation Sciences, Amman Arab University, Amman, 11953 Jordan; 4 Technical Faculty of Ferdows, University of Birjand, Birjand, 9717434765 Iran; 5 School of Electrical Engineering and Computer Science, KTH Royal Institute of Technology, 100-44 Stockholm, Sweden; 6 Department of Electrical and Computer Engineering, Science and Research Branch, Islamic Azad University, Tehran, 14778-93855 Iran; 7 School of Engineering and the Built Environment, Edinburgh Napier University, 10 Colinton Rd., Edinburgh, EH10 5DT UK; 8 Faculty of Electronics, Telecommunications and Informatics, Gdansk University of Technology, 80-233 Gdansk, Poland; 9 Engineering Optimization and Modeling Center, Reykjavik University, 101 Reykjavik, Iceland; 10 Electronic Engineering Department, University of Rome “Tor Vergata”, Via del Politecnico 1, 00133 Rome, Italy

**Keywords:** Engineering, Electrical and electronic engineering

## Abstract

A novel technique is shown to improve the isolation between radiators in antenna arrays. The proposed technique suppresses the surface-wave propagation and reduces substrate loss thereby enhancing the overall performance of the array. This is achieved without affecting the antenna’s footprint. The proposed approach is demonstrated on a four-element array for 5G MIMO applications. Each radiating element in the array is constituted from a 3 × 3 matrix of interconnected resonant elements. The technique involves (1) incorporating matching stubs within the resonant elements, (2) framing each of the four-radiating elements inside a dot-wall, and (3) defecting the ground plane with dielectric slots that are aligned under the dot-walls. Results show that with the proposed approach the impedance bandwidth of the array is increased by 58.82% and the improvement in the average isolation between antennas #1&2, #1&3, #1&4 are 8 dB, 14 dB, 16 dB, and 13 dB, respectively. Moreover, improvement in the antenna gain is 4.2% and the total radiation efficiency is 23.53%. These results confirm the efficacy of the technique. The agreement between the simulated and measured results is excellent. Furthermore, the manufacture of the antenna array using the proposed approach is relatively straightforward and cost effective.

## Introduction

The 5th generation mobile communication system brings numerous advantages over the 4G standard, such as higher throughput and shorter latency^1^. This is enabled by increasing the number of antenna array elements on both the transmitting and the receiving end, which can be achieved by using multiple-input–multiple-output (MIMO) antennas at sub-6 GHz band^2,3^. As the size of smartphones is relatively small, recently reported MIMO antenna array designs for future 5G mobile devices can only accommodate up to 12 array elements^4–6^. The space limitation implies tighter spacing between the radiating elements and, consequently, the requirement of high isolation between the radiators is a practical challenge^7–11^. This is because mutual coupling between the radiating elements in the array can have a detrimental effect the performance of MIMO in terms of impedance mismatching, radiation pattern deviation, increase in side-lobe level, scan blindness, high signal correlation, and low radiation spectral efficiency^12–16^.

Smartphones have a limited space and therefore the overall footprint of the MIMO antennas must be restricted too. To overcome this limitation, it is therefore unavoidable to tightly integrate the antennas in the array^17,18^. To improve the isolation between the array elements various techniques have been reported in the literature. In^19^ an eight-port dual-polarized antenna array is proposed for 5G applications. The array covers the 2.6 GHz band (2550–2650 MHz) with return-loss better than 10 dB, cross polarization discrimination better than 20 dB, and isolations between the antennas is of 12 dB. A tri-polarized twelve element antenna array in^20^ operates at the 3.5 GHz band (3.4–3.6 GHz) for future 5G MIMO smartphone. Mutual couplings are reduced by using a standard polarization technique. The array is reported to achieve a return-loss better than 10 dB, isolation between the array elements of 12.5 dB, and antenna efficiency higher than 50%. In^21^ a neutralization line technique is employed to suppress the electromagnetic coupling between the eight and sixteen closely spaced quad-linear arrays within the 3.4–3.6 GHz band. The average isolation between the array elements is limited to 10 dB. In^22^ two asymmetrically mirrored gap-coupled loop antennas are combined as a self-decoupled system to provide isolation between the antennas of 10 dB over 3.4–3.6 GHz. In^23^, a novel orthogonal-mode scheme is reported where the isolation of better than 17 dB is achieved between the closely spaced antenna elements over 3.4–3.6 GHz. Although the above techniques provide a means to implement high integration and acceptable levels of isolations, the decoupling property is achieved over a relatively narrow band that falls short to accommodate the entire 5G band. The challenge remains to be the development of a viable technique for smartphone MIMO systems that provides high isolation between radiators over the entire 5G spectrum^24^.

This paper presents an effective technique to mitigate inter-radiator coupling that can otherwise degrade the performance of MIMO antennas. It is shown that the proposed approach provides high isolation between the adjacent radiators, as well as an improvement in impedance matching, bandwidth, gain, and total radiation efficiency. This is achieved by loading resonant elements with a T-shaped matching load, framing the radiation elements inside decoupling dot-wall, and by defecting the ground plane with square slots that are aligned with the dot-walls. The proposed MIMO antenna comprises a 3 × 3 matrix of square ring-shaped resonators is designed to operate over the frequency range 3.0–5.5 GHz, which corresponds to a fractional bandwidth (FBW) of about sixty percent. Unlike conventional isolation improvement techniques reported to date, the proposed method provides a high average isolation, which is greater than 27 dB, between the radiating elements in the array. The proposed MIMO’s performance is validated by both simulation and measurements.

## High-performance antenna array design

This section describes the design process used for implementing the 4-element antenna array for 5G sub-6 GHz MIMO applications. Also, evaluated here is the array’s performance.

### The reference four-element antenna array

**Figure 1:**
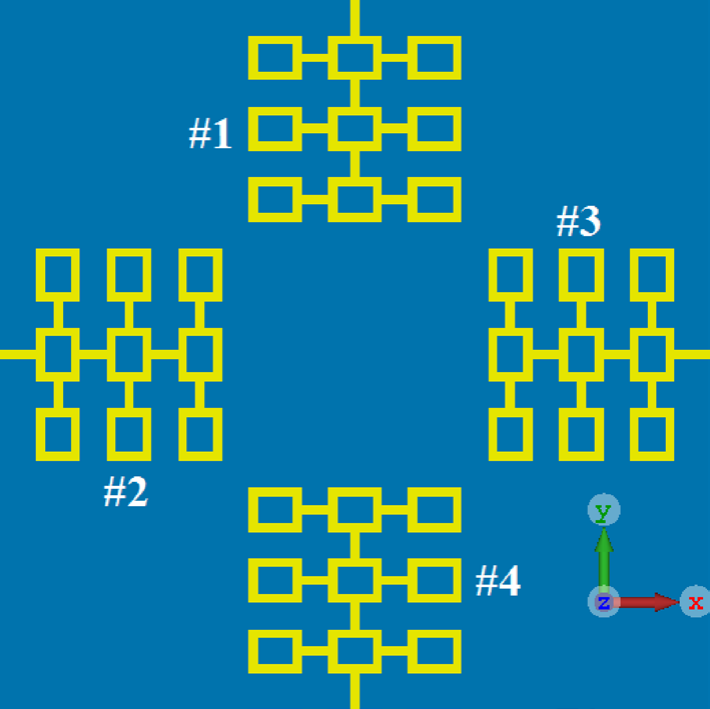
Top view showing the layout of the proposed antenna array consisting of four radiating elements with a standard ground plane.

The layout of the reference antenna array is shown in Fig. 1. It is constructed from four radiating elements implemented in a symmetric configuration. Each radiation element comprises 3 × 3 matrix of square ring-shaped resonators, where the central resonator of each row is interconnected to each other and the feedline. The dimensions of the ring were optimized using the optimization tool in CST Microwave Studio, which is a 3D full-wave electromagnetic solver. The antenna array was fabricated on the Rogers RT5880 lossy substrate with dielectric constant of *ε*_*r*_ = 2.2, tanδ = 0.0009, and thickness of *h* = 0.6 mm.

**Figure 2:**
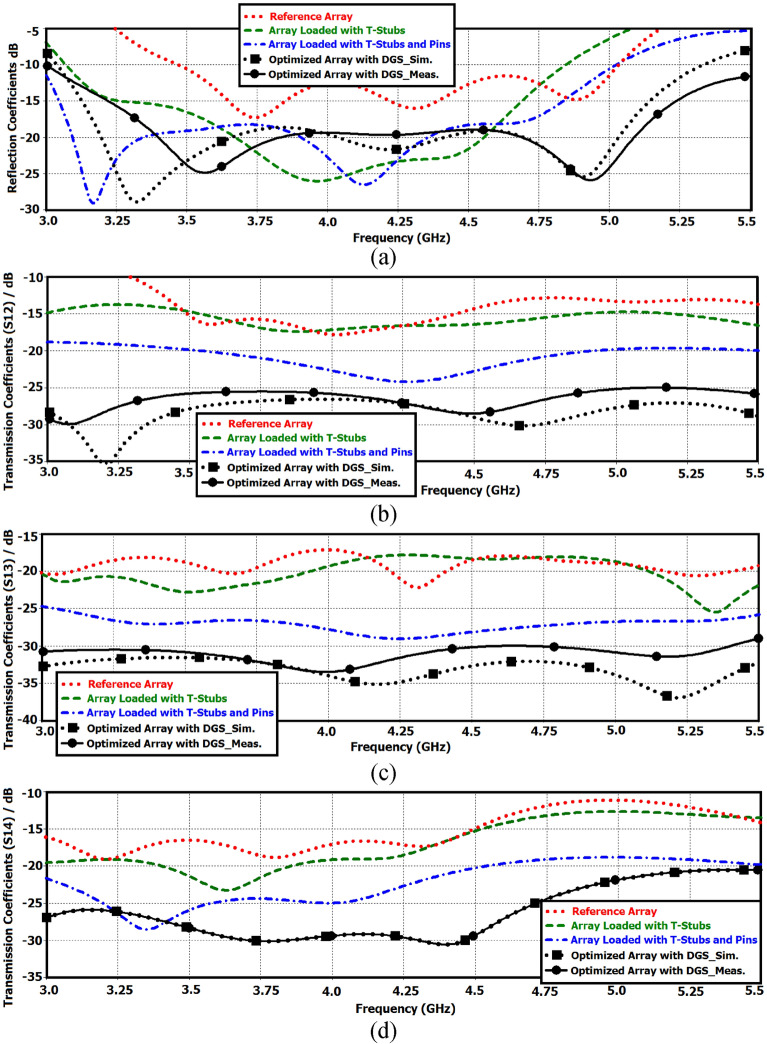
Comparison of the reflection and transmission coefficient responses of the reference antenna array with application of various techniques: (**a**) reflection coefficient response, (**b**) transmission coefficient (*S*_12_) between antennas #1&2, (**c**) transmission coefficient (*S*_13_) between antennas #1&3, and (**d**) transmission coefficient (*S*_14_) between antennas between antennas #1&4.

Each antenna in the array can be separately excited through a microstrip feedline. Dimensions of the rings, the gap between the interconnected rings, and each radiating element are 6 × 5 mm^2^, 3 mm, and 25 × 25 mm^2^, respectively. The overall dimension of the 4-element array is 80 × 80 × 0.6 mm^3^. The length of each feedline in the prototype antenna is 7 mm. The feedline length can be reduced with no implication of the antenna’s performance. With reduced feedline the overall antenna size can be reduced to 70 × 70 mm^2^, which can be easily accommodated in various smartphones. As the signal wavelength is inversely related to the square root of dielectric constant, the overall antenna size can be further reduced by constructing the array on a substrate with a high dielectric constant. Figure 2 shows the reflection (S_11_) and the transmission coefficient (S_12_, S_13_ and S_14_) responses of the reference array. As the array configuration is symmetrical the reflection and transmission coefficient responses are identical for other array ports. The results show that the array has a fraction bandwidth of 35.29% with an average impedance match of 12 dB across the entire sub-6 GHz band (3.5–5 GHz). The average isolation between antennas #1&2, #1&3, and #1&4 are 13 dB, 17 dB, and 15 dB, respectively. Although the magnitude of the isolation is satisfactory however to ensure a low envelope correlation coefficient (ECC) and therefore higher data rate transmission the isolation needs to be more robust.

In the next two sections, three new yet simple techniques are described to improve the impedance matching and impedance bandwidth, and to suppress the mutual coupling between the radiating elements in the array.

### Four-element antenna array loaded with T-shaped matching stubs

**Figure 3:**
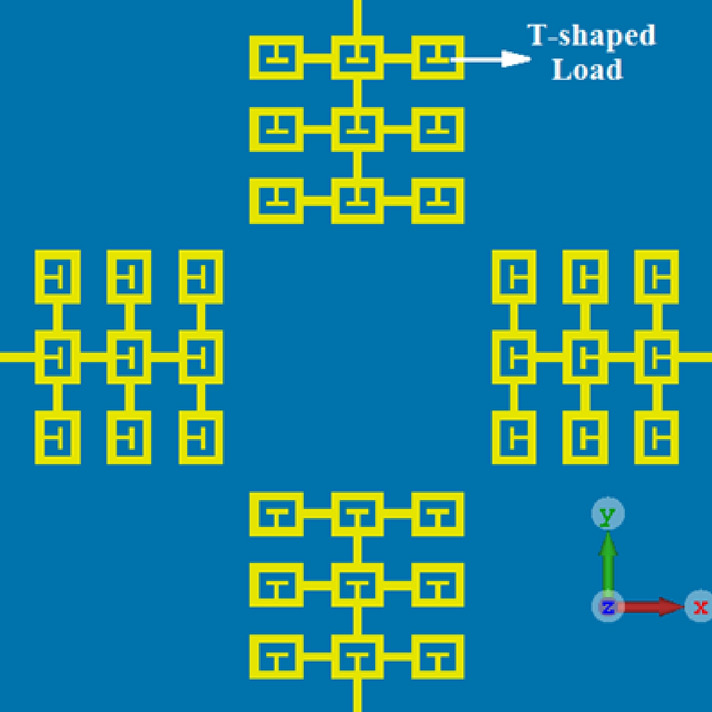
Reference antenna array loaded with T-shaped matching stubs. The standard ground plane remains unchanged.

To improve the impedance matching and impedance bandwidth of the reference antenna array a novel, yet simple technique is applied to it that preserves the array’s physical footprint. This is achieved by loading the interconnected square ring resonators with T-shaped matching stubs, as shown in Fig. 3. The simulation results in Fig. 2 show with the introduction of the stub the overall performance of the array is improved. The improvement is attributed to reduction of the surface-wave interaction between the four radiators. By incorporating the T-shaped stubs the fractional bandwidth for |S_11_|≤ − 10 dB increases to 44.02% across 3.1–4.85 GHz, which corresponds to an improvement of ~ 10% compared to the reference array. Moreover, on average there is 8 dB improvement in the impedance matching over the 3.1–4.85 GHz band, and the improvement in the isolation between the four radiators in the array is approximately 3 dB. Across 3.5–5 GHz the average impedance matching is 20 dB, and the average isolation between antennas #1&2, #1&3, and #1&4 are 16 dB, 19 dB, and 18 dB, respectively.

In the next section, further improvement is introduced to the T-loaded antenna array.

### 4-Element antenna array loaded with T-shaped matching stubs and decoupling dot walls

**Figure 4:**
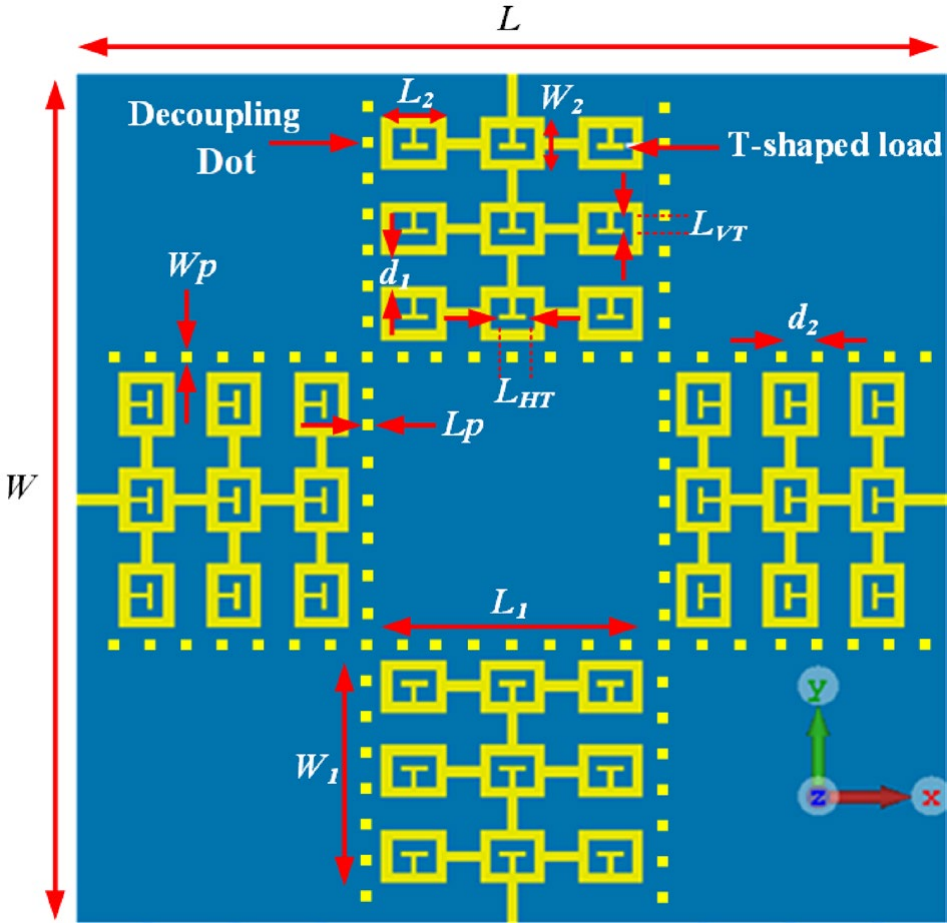
Proposed antenna array loaded with T-shaped matching stubs and decoupling dot-walls. The magnitude of the annotated geometrical parameters is listed in Table 1.

A major limiting factor that MIMO systems are plagued with is unwanted mutual coupling which arises mainly due to the smaller spacing between multiple antennas. Unwanted mutual coupling is the one of the parameters that degrades the channel capacity. In this section the unwanted mutual coupling between the array elements is reduced to improve the array’s data processing capability. This is achieved here by inserting linear arrangement of square-shaped dot-walls. The dot-walls are implemented on the substrate surface around each radiating element, as shown in Fig. 4, to suppress surface wave interaction between the radiators. As per substrate integrated waveguide the periodicity of the dots needs to be shorter than the wavelength of the highest frequency of the operating range of the array, and the dimension of the square dots needs to be a fifth of the highest frequency.

With application of the decoupling dot-walls the T-loaded array’s reflection and transmission coefficient responses in Fig. 2 shows the array’s fractional bandwidth for |S_11_|≤ − 10 dB improves to 50% (3–5 GHz), which is an improvement of ~ 15% compared to just the T-loaded array. The array has an average impedance matching of 21 dB, which is 9 dB greater than the reference array. The average isolation between antennas #1&2, #1&3, and #1&4 is 23 dB, 27 dB, 23 dB, respectively. Compared to the reference array this is an improvement of 10 dB, 10 dB, and 8 dB, respectively. These results confirm the effectiveness of the proposed decoupling approach.

In the next section the array’s performance is further improved by defected the ground plane.

### Four-element array antenna with defected ground plane

**Figure 5:**
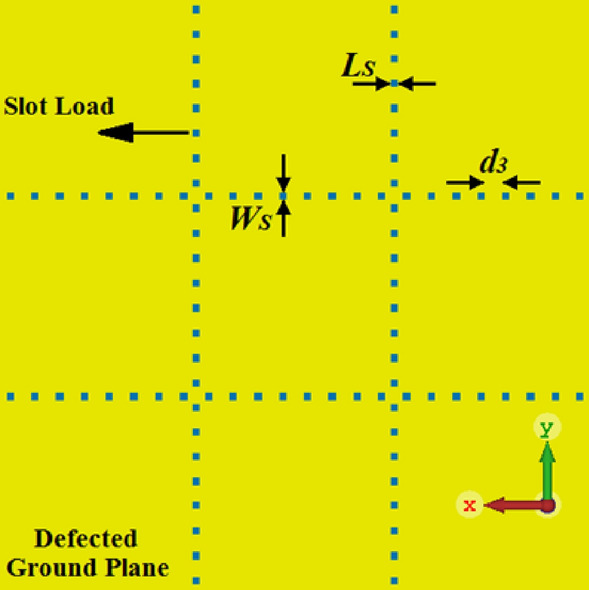
Defected ground plane with square slots. The top side of the array is same as in Fig. 4. Geometrical parameters of the array are given in Table 1.

In this section, the defected ground plane (DGP) technique is applied to the array’s ground plane to extend its operational bandwidth. The ground plane is defected with square shaped slots, as shown in Fig. 5. The slots are located between the dot-walls. The gap between the slots is shorter than the wavelength of the highest frequency of the operating range of the array, and the dimension of the square slot is fifth of the highest frequency. The slots eliminate surface currents between the antenna and the ground plane thereby reducing power loss and unwanted coupling between neighboring antennas.

The effect of DGP is compared with the previous structural iterations in Fig. 2. It is observed from Fig. 2 that application of slots on the ground plane of the array results in significant improvement in the array’s bandwidth and impedance matching performance. The structural parameters of the array that are annotated in Fig. 4 were optimized using CST Microwave Studio. The measured fractional bandwidth of the optimized array for |S_11_|≤  − 10 dB is 58.82% from 3.0 to 5.5 GHz. This is an improvement of 23.53% compared to the reference array. The average impedance match obtain improves to 20 dB. The isolation between antennas #1&2, #1&3, and #1&4 are 27 dB, 33 dB, and 28 dB, respectively. Compared to the reference array this is an improvement of 14 dB, 16 dB, and 13 dB, respectively. Geometrical design parameters of the proposed antenna array are listed in Table 1.

**Table 1:** Units for magnetic properties geometrical design parameters of the array antenna.

Dimensions of the substrate (*L* × *W*)	80 × 80 mm^2^
Dimensions of the antenna (*L*_*1*_ × *W*_*1*_)	25 × 25 mm^2^
Dimensions of the square rings (*L*_*2*_ × *W*_*2*_)	6 × 5 mm^2^
Vertical length of the T-shaped matching load (*L*_*VT*_)	1.5 mm
Horizontal length of the T-shaped matching load (*L*_*HT*_)	2.5 mm
Width of the T-shaped matching load (*W*_*T*_)	0.5 mm
Distance between the rings (*d*_*1*_)	3 mm
Dimension of the decoupling square dot (*L*_*P*_ × *W*_*P*_)	1 × 1 mm^2^
Distance between the decoupling dots (*d*_*2*_)	2 mm
Dimensions of the DGP's slots (*L*_*S*_ × *W*_*S*_)	1 × 1 mm^2^
Distance between the DGP's slots (*d*_*3*_)	2.35 mm
Thickness of the Rogers RT5880 substrate	0.6 mm

**Figure 6:**
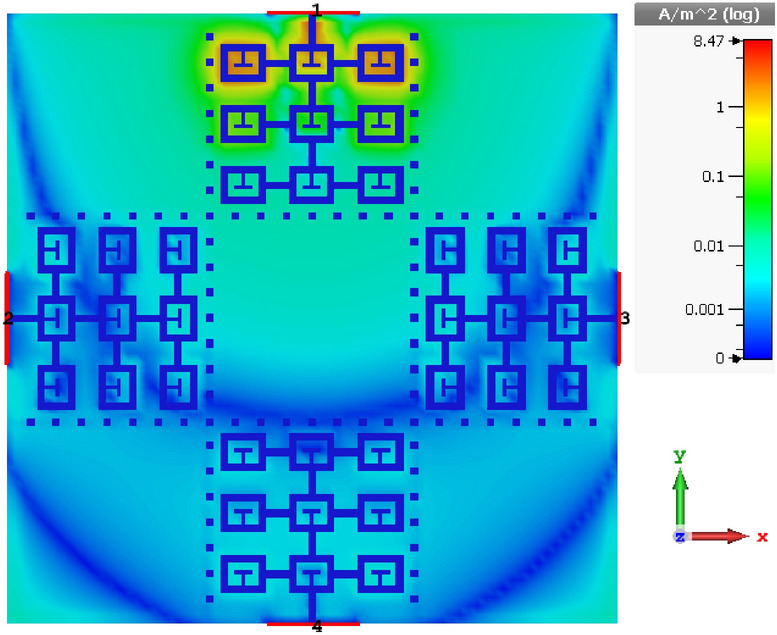
Current density over the proposed antenna array at 4.25 GHz.

The surface current density distribution over the proposed antenna array loaded with T-stubs and dot-walls, and with defected ground plane is shown in Fig. 6. It can be observed from this figure that there is virtually no interaction between the four radiation elements in the array. This demonstrates the effectiveness of the proposed technique.

**Figure 7:**
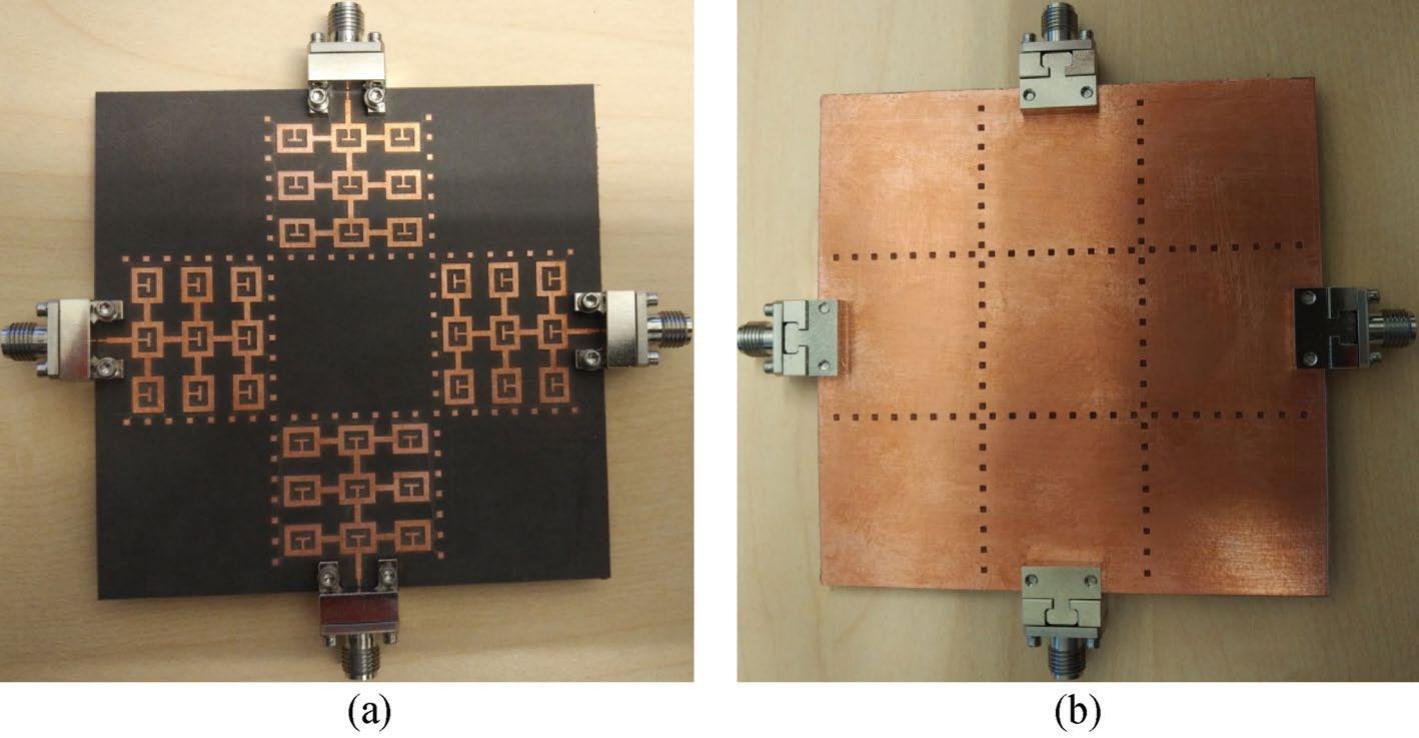
Fabricated prototype of the proposed four-element antenna array, (**a**) top side showing each square resonant ring is loaded with a T-shaped matching stub and each radiating element is enclosed within the isolating dot-walls, and (**b**) bottom side showing the defected ground plane loaded with square shaped slots that are aligned underneath the dot-walls.

The S-parameters of (1) the reference array, (2) reference array loaded with T-stub, (3) reference array loaded with T-stub and dot-walls, and (4) reference array loaded with T-stub and dot-walls array and with DGP are summarized in Table 2. The fabricated prototype of the optimized antenna array is shown in Fig. 7.

**Table 2:** S-Parameter comparison of the four antenna arrays.

S-parameters	|S_11_|< − 10 dB (GHz)	Ave. impedance matching (dB)	Ave. S_12_ (dB)	Ave. S_13_ (dB)	Ave. S_14_ (dB)	Fractional BW (%)
Reference antenna array	3.5 – 5.0	12	13	17	15	35.29
Array with T-stub	3.1—4.85	20	16	19	18	44.02
Array with T-stub and dot-wall	3.0—5.0	21	23	27	23	50
Optimized Array with DGS (measured)	3.0—5.5	20	27	33	28	58.82
Improvement of optimized array c.f. reference array	1	8	14	16	13	23.53

### Lumped element equivalent circuit model of the antenna array

**Figure 8:**
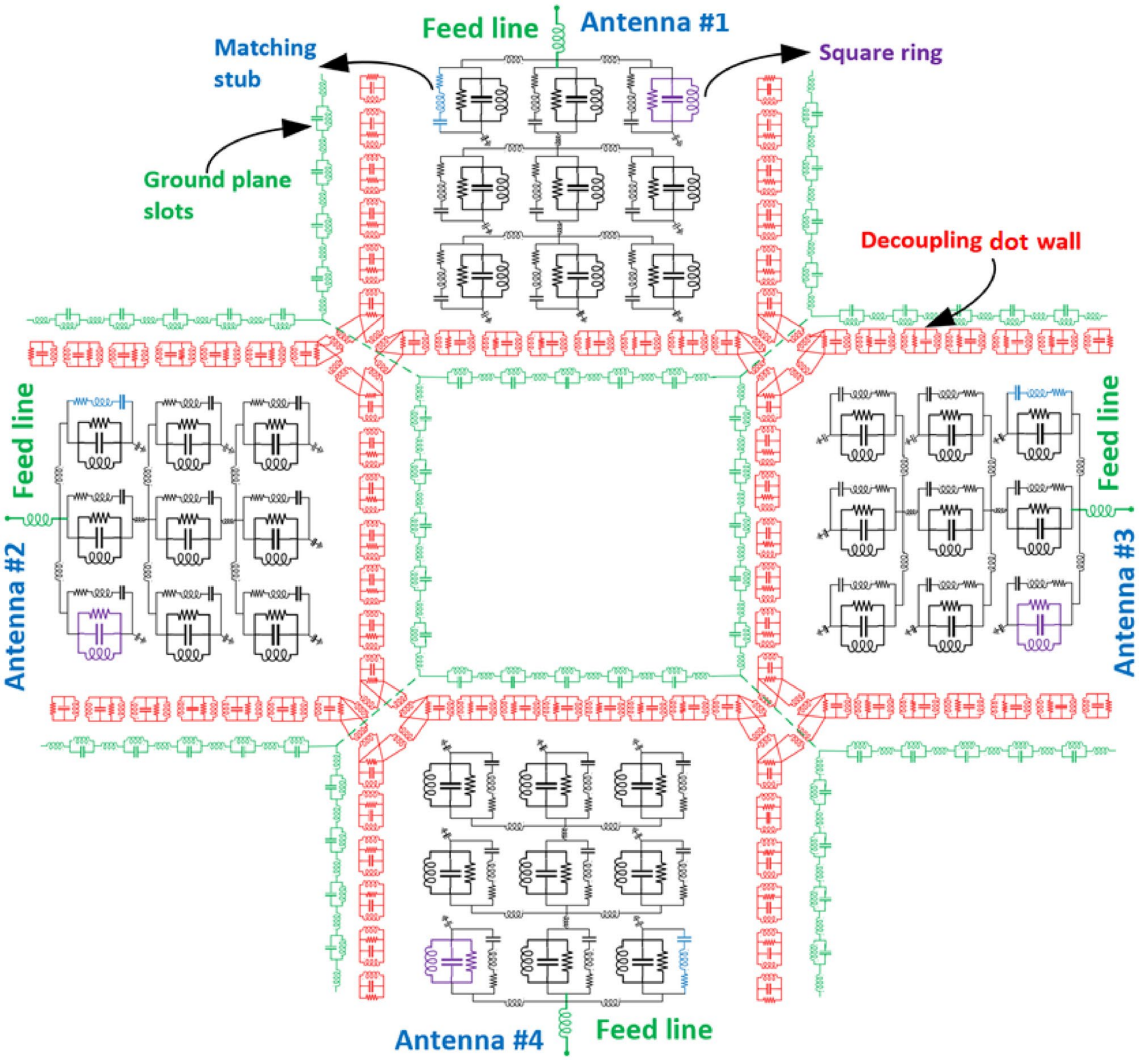
Equivalent circuit lumped element model of the proposed antenna array.

The equivalent circuit lumped element model of the proposed antenna array is depicted in Fig. 8. Each of the four-radiating elements in the array consists of square ring resonators represented by parallel *RLC* lumped element components, and the matching T-shaped stub is represented by series *RLC* components. The substrate loss is represented by the resistive component. The decoupling dot-wall is represented inductively coupled *RLC* components. The decoupling dot-walls surround each of the four radiating elements in the array. Ground plane slots are represented by a configuration consisting of parallel *LC* and series *L* components that are serially interconnected to each other^25^. The ground plane slots are aligned under the decoupling dot-wall.

### Radiation characteristics

**Figure 9:**
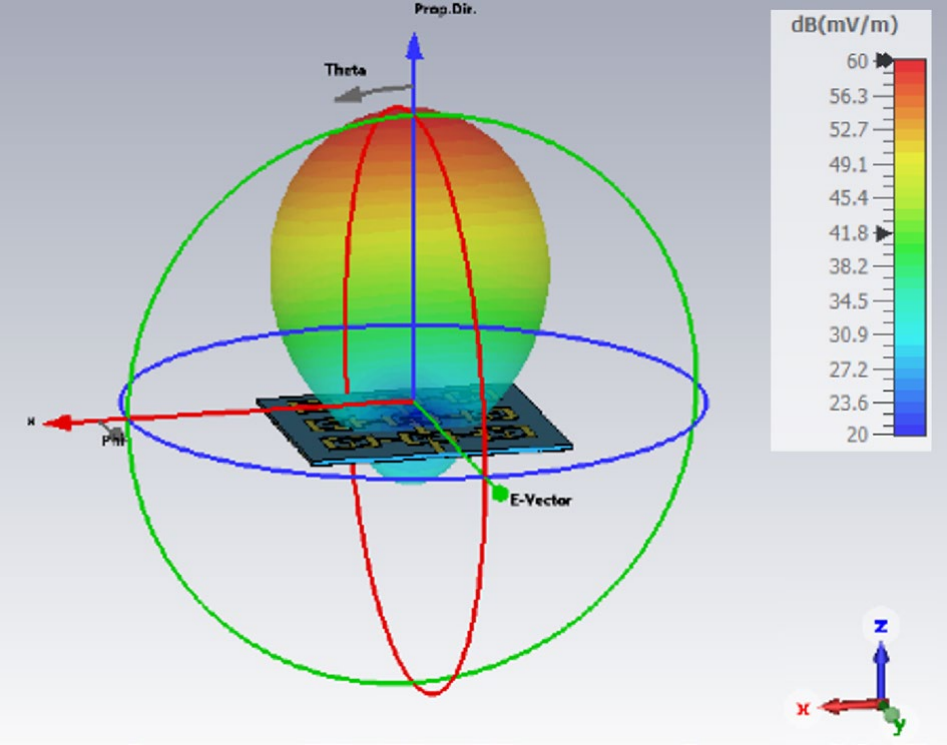
Simulated radiation pattern of the single antenna array.

**Figure 10:**
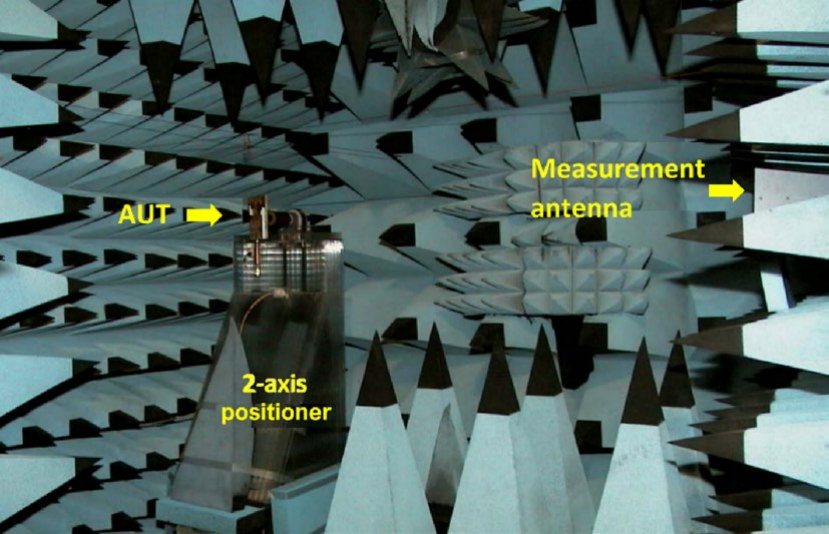
Antenna measurement setup in anechoic chamber.

**Figure 11:**
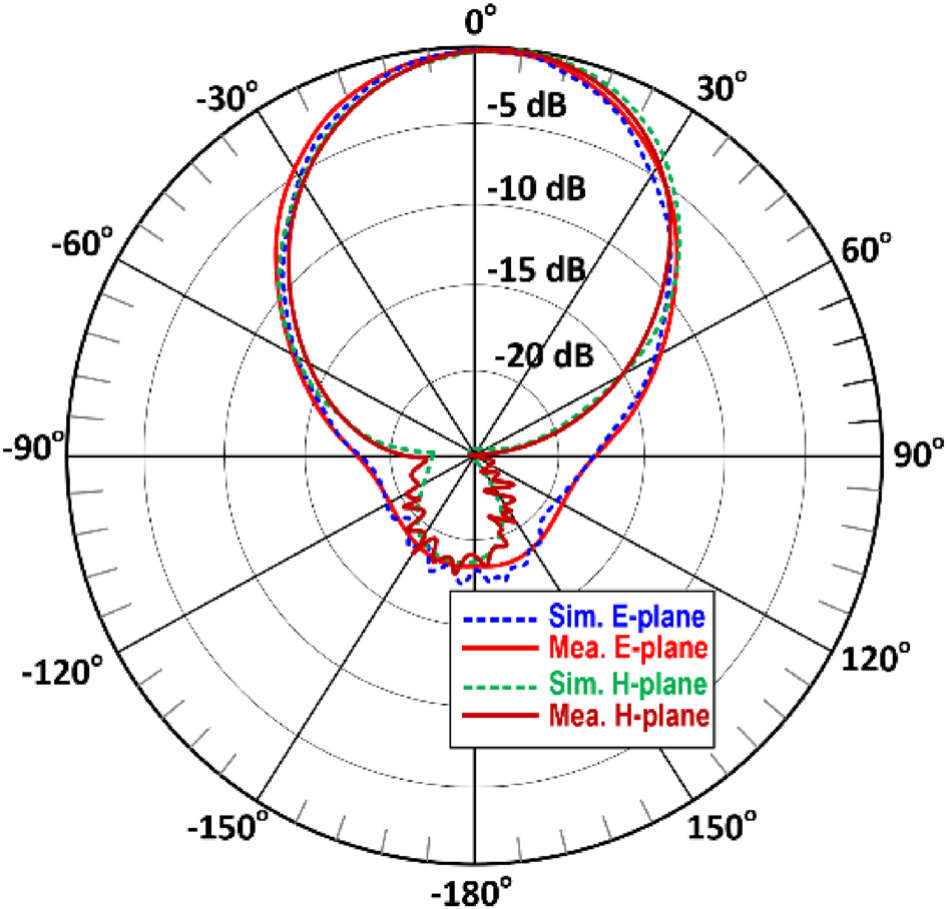
Normalized simulated and measured radiation pattern of the single antenna in E and H-planes at 4.25 GHz.

The simulated radiation pattern of the single antenna in the array using CST Microwave Studio is shown in Fig. 9. The broadside antenna is highly directional. Photograph of the measurement setup is shown in Fig. 10. The floor and ceiling of the anechoic chamber are lined with radiation absorbent material (RAM) to prevent the reflections from affecting the measurements. A fixed wideband horn antenna was the measurement antenna. The antenna array, i.e., antenna under test (AUT), is rotated about two axes, and its movements are computer controlled. The measured and simulated radiation patterns of the antenna in the E- and H-planes at 4.25 GHz is shown in Fig. 11.

**Figure 12:**
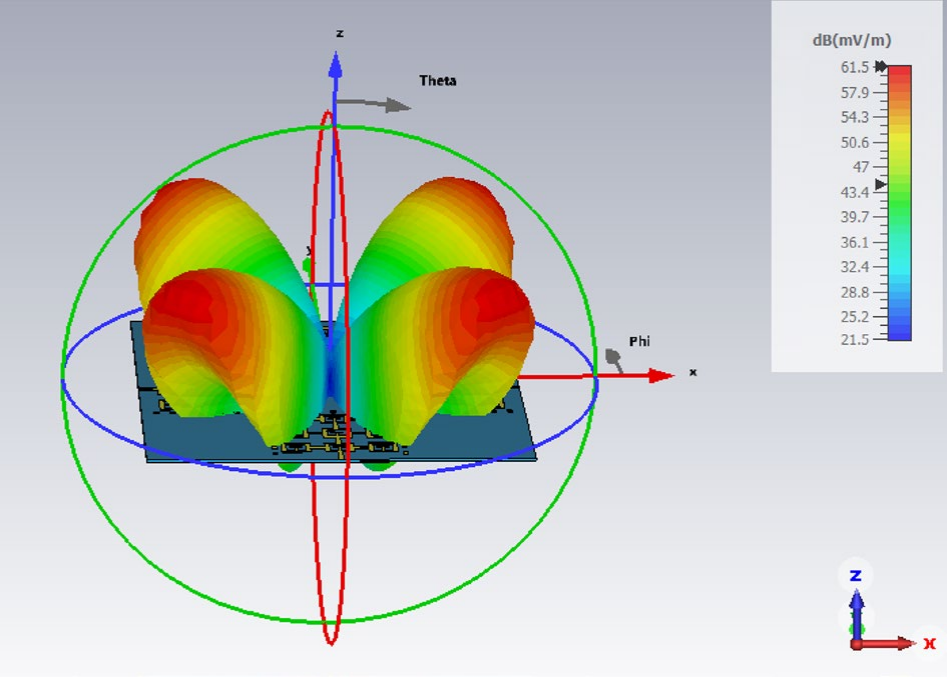
Simulated radiation pattern of the proposed 4-element antenna array at 4.25 GHz.

**Figure 13:**
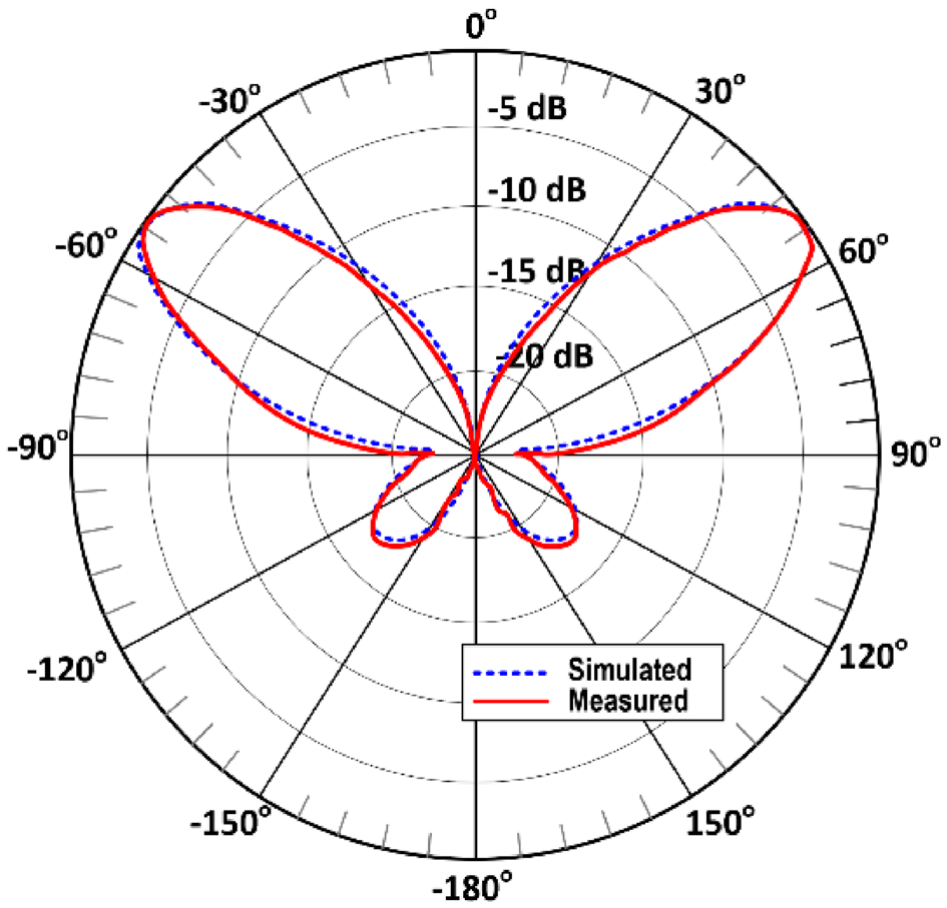
Normalized simulated and measured radiation pattern (E and H-planes) of the proposed 4-element antenna array at 4.25 GHz.

The simulated radiation pattern of the proposed four element antenna array is shown in Fig. 12 when all four antennas are excited simultaneously. The array radiates highly directional beams at angles of ± 45° and ± 135° in the azimuth plane. The measured and simulated radiation patterns of the array in the E- and H-planes at 4.25 GHz shown in Fig. 13 are identical. There is good agreement between the measured and simulated radiation patterns.

**Figure 14:**
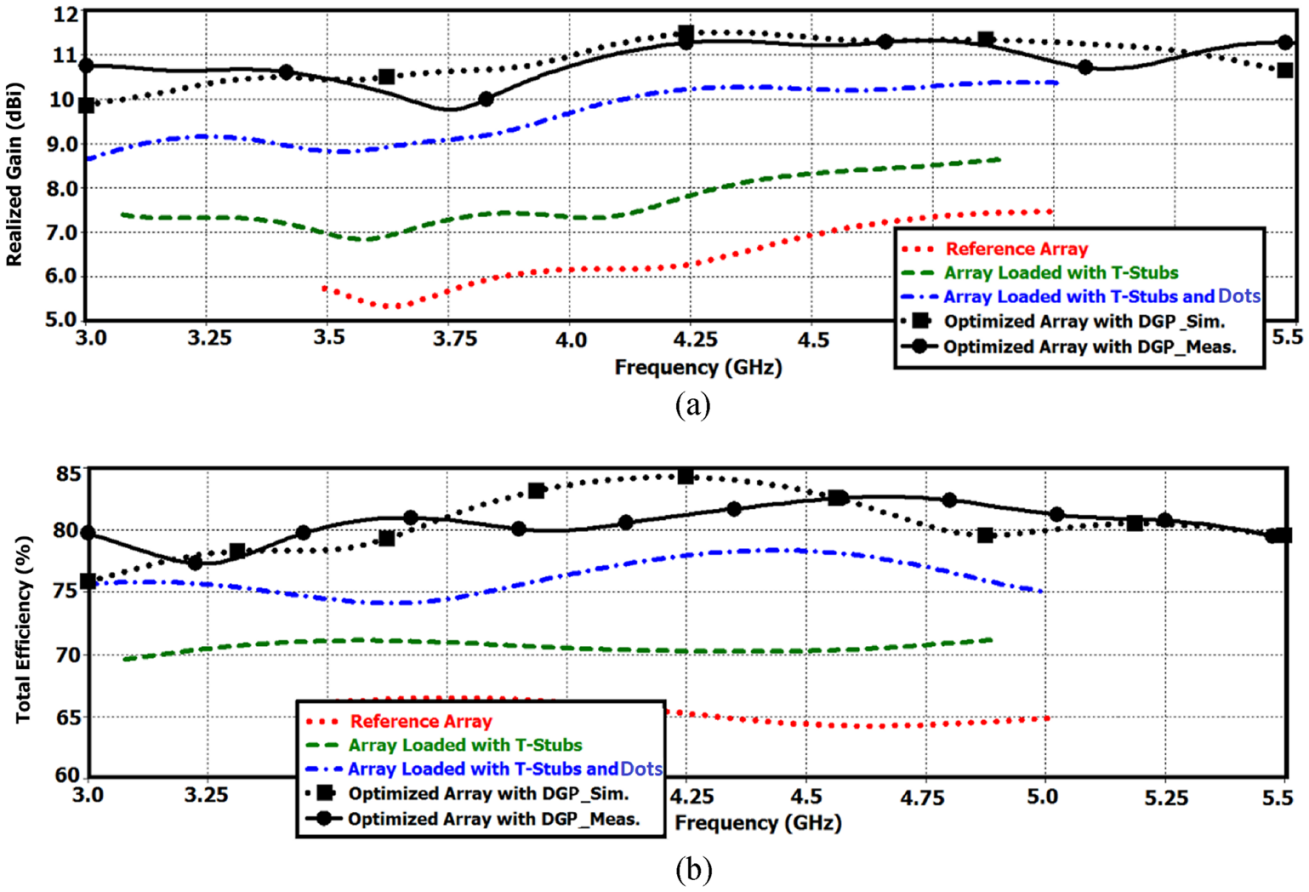
Radiation properties of the proposed 4-element antenna array, (**a**) realized gain, and (**b**) total efficiency.

The radiation characteristics, specifically, gain and total efficiency of the various antenna arrays investigated in this study are shown in Fig. 14. Gain method was used here to measure the radiation efficiency using the expression *η* = G/D, where *G* is the gain of the antenna array measured using a standard gain horn, and *D* is the directivity of array. Directivity was determined from the expression *D* = 41,253/ΔθΔϕ, where Δθ and Δϕ are the principal plane beamwidths (in decimal degrees) measured from the radiation pattern of the antenna as described in^26^.

In Fig. 14a the realized gain of the (1) reference array, (2) reference array loaded with T-stubs, (3) reference array loaded with T-stubs and dot-walls, and (4) reference array loaded with T-stubs and dot-walls and DGS is observed to vary over 5.25–7.5 dBi, 6.9–8.65 dBi, 8.8–10.35 dBi, and 9.8–11.25 dBi, respectively. The average gain of the optimized antenna array is 4.2 dB larger than the reference array, which demonstrates the effectiveness of the proposed design approach.

The total efficiency of the various iterations made to the reference array are shown in Fig. 14b. The efficiency of the reference array, reference array loaded with T-stubs, reference array loaded with T-stubs and dot-walls, and reference array loaded with T-stubs and dot-walls and DGS is shown to vary over 63–65.5%, 69.5–71.5%, 74–78%, and 78–84%, respectively. Compared to the reference array the improvement in the efficiency of the optimized array is 17%. The gain and efficiency performance of the various arrays is summarized in Tables 3 and 4.

**Table 3:** Antenna array gain.

Gain	Minimum value	Maximum value	Average value (dBi)
Ref. Ant	5.25 dBi @ 3.62 GHz	7.5 dBi @ 5.0 GHz	6.4
Ant. with T-stub	6.9 dBi @ 3.56 GHz	8.65 dBi @ 4.85 GHz	7.75
Ant. with T-stub and dot-wall	8.8 dBi @ 3.0 GHz	10.35 dBi @ 5.0 GHz	9.6
Optimized Ant. with DGS (Measured)	9.8 dBi @ 3.75 GHz	11.25 dBi @ 4.35 GHz	10.6
Average improvement of optimized Ant. c.f. Ref. Ant	4.2 dBi

**Table 4:** Antenna array total efficiency.

Total Efficiency	Minimum value	Maximum value	Average value (%)
Ref. Ant	63% @ 4.62 GHz	65.5% @ 3.8 GHz	64
Ant. with T-stub	69.5% @ 3.12 GHz	71.5% @ 3.6 GHz	70
Ant. with T-stub and dot-wall	74% @ 3.62 GHz	78% @ 4.4 GHz	76
Optimized Ant. with DGS (Measured)	78% @ 3.25 GHz	84% @ 4.65 GHz	81
Average improvement of optimized Ant. c.f. Ref. Ant	17%		

Envelope correlation coefficient (*ECC*) is a major metric to characterize MIMO antennas. It indicates the correlation between the radiating antenna elements, and it directly impacts the channel capacity of the wireless system. *ECC* is defined by^27^

1 $$ECC = \frac{{\left| {\iint_{4\pi } {E_{1} (\theta ,\phi )*E_{2} (\theta ,\phi )d\Omega }} \right|^{2} }}{{\iint_{4\pi } {\left| {E_{1} (\theta ,\phi )} \right|^{2} d\Omega \iint_{4\pi } {\left| {E_{2} (\theta ,\phi )} \right|^{2} d\Omega }}}}$$

where *E*_*1*_ and *E*_*2*_ are far-field radiation patterns of antenna #1 and antenna #2, respectively. *ECC* can be determined from S-parameters measurements using^28^

2 $$ECC = \frac{{\left| {S_{11}^{*} S_{12} + S_{22}^{*} S_{21} } \right|^{2} }}{{\left[ {1 - \left( {\left| {S_{11} } \right|^{2} + \left| {S_{21} } \right|^{2} } \right)} \right]\left[ {1 - \left( {\left| {S_{22} } \right|^{2} + \left| {S_{12} } \right|^{2} } \right)} \right]}}$$

**Figure 15:**
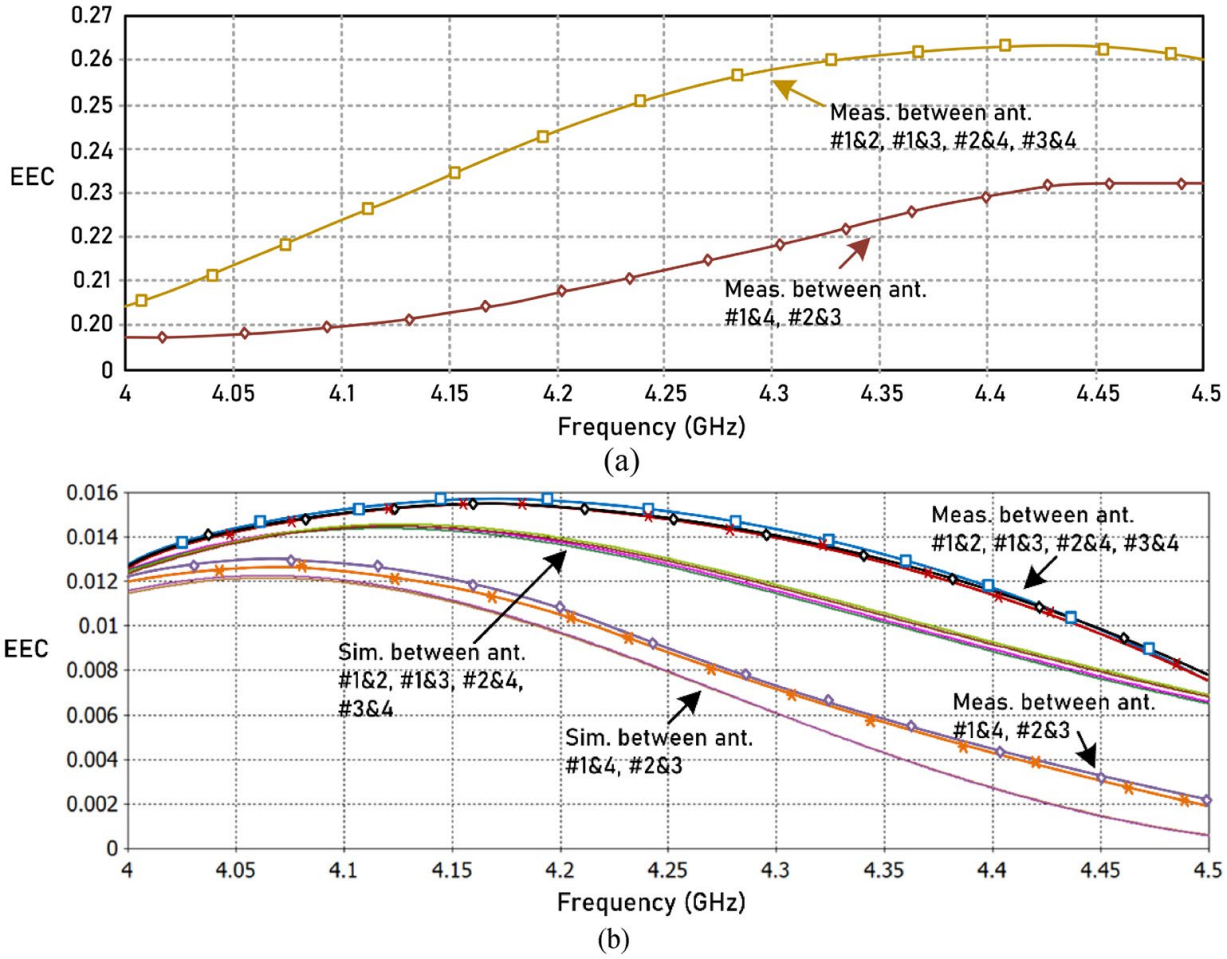
(**a**) Measured ECC between the four antennas for the 4-element reference array, and (**b**) simulated and measured ECC between the four antennas in the proposed 4-element antenna array.

The ideal value of *ECC* is zero, however, in practical applications an *ECC* < 0.5 is acceptable. Figure 15 shows how the *ECC* varies over the operating frequency range of the reference array and proposed array. Measurement results show compared to the reference array the correlation between the antennas in the array over its operating frequency range of the proposed array is significantly reduced. The correlation of the proposed array is < 0.016. In fact, the correlation between opposite antennas (#1&4 & #2&3) is < 0.013. The correlation between adjacent antennas tends to reduce for frequencies above 4.18 GHz, and for opposite antennas it reduces for frequencies above 4.07 GHz. In MIMO systems the antenna array is used for diversity reception. The degree of reduction in transmission power in a diversity scheme is determined by the diversity gain (*DG*), which is calculated using^28^

3 $$DG = 10\sqrt {1 - ECC}$$

Using the worst-case *ECC* value of the proposed array in Fig. 15 the *DG* is > 9.9 dB. This confirms an excellent diversity performance by the proposed array which makes it suitable for high data rate transmission.

## State-of-the-art comparison

The performance of the proposed antenna array is compared in Table 5 with some recent state-of-the-art array designs that have been reported in the literature. Compared to other design approaches cited in the table the isolation achieved with the proposed array is the highest. Only reference^29^ provides information on the gain. Compared to^29^ which has a maximum gain of 2 dBi the proposed antenna has a significantly higher gain of 11.25 dBi. The proposed decoupling approach is less complex compared with^30,31^. Also, the proposed approach has the largest fractional bandwidth than other arrays cited in the table. However, the efficiency of the proposed array is comparable to other arrays. These features make the proposed antenna array suitable for sub-6 GHz 5G MIMO communication systems.

**Table 5:** State-of-the-art comparison.

Ref	Design approach	Fractional bandwidth (%)	Isolation between radiators (dB)	Max. gain (dBi)	Max. eff. (%)	Design complexity	MIMO application
^19^	Orthogonal polarization	3.8	> 12	–	–	Low	Yes
^20^	Tri-polarized	5.7	> 10	–	70	Low	Yes
^29^	T-shaped slot	5.7	> 11	2	90	Low	Yes
^30^	Self-decoupled	246	> 7	–	85.1	Moderate	Yes
^31^	Multi-slot decoupling	12.2/15.4	> 15.5	–	48/80	High	Yes
^32^	Decoupling network	19.5/51.1	> 15	–	–	Low	Yes
^33^	Decoupling stub	24/4	> 11.5	–	76.5/79	Low	Yes
This work	Matching stubs + dot-wall + DGP	58.82	> 27	11.25	84	Low	Yes

## Conclusion

The design of a novel four-element antenna array is shown to satisfy the requirements for high performance sub-6 GHz 5G MIMO communication systems. The array’s performance has been verified experimentally. The array design comprises four radiation elements in a cross shaped configuration. Each radiating element consists of three rows of interconnected square ring resonators embedded in which is a T-shaped matching stub. The four radiating elements are framed inside dot-walls. The ground plane is defected with dielectric slots that are aligned under the dot-walls. This approach is shown to enhance the arrays impedance bandwidth, impedance matching, and isolation between the radiating elements. Moreover, the correlation between the radiating antenna elements is extremely low. This confirms the channel capacity in MIMO systems is unaffected and excellent diversity performance is achieved. These attributes make the array suitable for sub-6 GHz 5G MIMO systems for high data rate transmission.

## Data Availability

All data generated or analyzed during this study are included in this article. All of the figures, materials, and data within the manuscript are original and owned by authors.
